# Impact of a Telehealth Program With Voice Recognition Technology in Patients With Chronic Heart Failure: Feasibility Study

**DOI:** 10.2196/mhealth.7058

**Published:** 2017-10-02

**Authors:** Heesun Lee, Jun-Bean Park, Sae Won Choi, Yeonyee E Yoon, Hyo Eun Park, Sang Eun Lee, Seung-Pyo Lee, Hyung-Kwan Kim, Hyun-Jai Cho, Su-Yeon Choi, Hae-Young Lee, Jonghyuk Choi, Young-Joon Lee, Yong-Jin Kim, Goo-Yeong Cho, Jinwook Choi, Dae-Won Sohn

**Affiliations:** ^1^ Department of Internal Medicine Seoul National University College of Medicine Seoul Republic Of Korea; ^2^ Healthcare System Gangnam Center Seoul National University Hospital Seoul Republic Of Korea; ^3^ Cardiovascular Center Seoul National University Hospital Seoul Republic Of Korea; ^4^ Office of Hospital Information Seoul National University Hospital Seoul Republic Of Korea; ^5^ Cardiovascular Center Seoul National University Bundang Hospital Seongnam Republic Of Korea; ^6^ Department of Cardiology Asan Medical Center University of Ulsan College of Medicine Seoul Republic Of Korea; ^7^ AIMMED Co., Ltd. Seoul Republic Of Korea; ^8^ Department of Biomedical Engineering Seoul National University College of Medicine Seoul Republic Of Korea

**Keywords:** heart failure, telemedicine, selfcare, compliance

## Abstract

**Background:**

Despite the advances in the diagnosis and treatment of heart failure (HF), the current hospital-oriented framework for HF management does not appear to be sufficient to maintain the stability of HF patients in the long term. The importance of self-care management is increasingly being emphasized as a promising long-term treatment strategy for patients with chronic HF.

**Objective:**

The objective of this study was to evaluate whether a new information communication technology (ICT)–based telehealth program with voice recognition technology could improve clinical or laboratory outcomes in HF patients.

**Methods:**

In this prospective single-arm pilot study, we recruited 31 consecutive patients with chronic HF who were referred to our institute. An ICT-based telehealth program with voice recognition technology was developed and used by patients with HF for 12 weeks. Patients were educated on the use of this program via mobile phone, landline, or the Internet for the purpose of improving communication and data collection. Using these systems, we collected comprehensive data elements related to the risk of HF self-care management such as weight, diet, exercise, medication adherence, overall symptom change, and home blood pressure. The study endpoints were the changes observed in urine sodium concentration (uNa), Minnesota Living with Heart Failure (MLHFQ) scores, 6-min walk test, and N-terminal prohormone of brain natriuretic peptide (NT-proBNP) as surrogate markers for appropriate HF management.

**Results:**

Among the 31 enrolled patients, 27 (87%) patients completed the study, and 10 (10/27, 37%) showed good adherence to ICT-based telehealth program with voice recognition technology, which was defined as the use of the program for 100 times or more during the study period. Nearly three-fourths of the patients had been hospitalized at least once because of HF before the enrollment (20/27, 74%); 14 patients had 1, 2 patients had 2, and 4 patients had 3 or more previous HF hospitalizations. In the total study population, there was no significant interval change in laboratory and functional outcome variables after 12 weeks of ICT-based telehealth program. In patients with good adherence to ICT-based telehealth program, there was a significant improvement in the mean uNa (103.1 to 78.1; *P*=.01) but not in those without (85.4 to 96.9; *P*=.49). Similarly, a marginal improvement in MLHFQ scores was only observed in patients with good adherence (27.5 to 21.4; *P*=.08) but not in their counterparts (19.0 to 19.7; *P*=.73). The mean 6-min walk distance and NT-proBNP were not significantly increased in patients regardless of their adherence.

**Conclusions:**

Short-term application of ICT-based telehealth program with voice recognition technology showed the potential to improve uNa values and MLHFQ scores in HF patients, suggesting that better control of sodium intake and greater quality of life can be achieved by this program.

## Introduction

Heart failure (HF) is a major public health issue, with a prevalence of over 23 million worldwide and rising with the aging of the population [1,2]. The characteristic feature of HF is a progressive loss of cardiomyocytes and the development of cardiac dysfunction, ultimately leading to frequent hospitalization and significant morbidity and mortality [1,3-5]. There are ongoing efforts to develop improved therapeutic and preventive strategies for HF, as most current strategies fail to reduce readmission rates and maintain the stability of HF patients in the long term [6,7].

Most hospitalizations for acute decompensated HF are attributable to poor self-care, including lack of knowledge, nonadherence to proper diet or medications, and failure of self-management of symptoms. Recent studies on patient-centered out-of-hospital management of HF patients have shown improvements in prognosis and quality of life [3,8-10]. However, in practice, barriers to self-care management such as insufficient personalized real-time feedback to patients regarding body weight, fluid balance, and physical activity limit their effectiveness [8,11,12]. Frequent assessment of physiologic parameters related to HF aggravation and remote disease management aided by advances in telemonitoring systems based on information communication technology (ICT) may be an approach to overcome the limitations of self-management. By enabling early detection of HF decompensation and through timely intervention, it may be possible to achieve improved outcomes and reduced medical costs for HF patients [11,13].

Voice recognition technology enables the recognition and translation of verbal information into text, which can then be used in automated data processing systems. With advances in the accuracy of this technology, it is being applied in the medical field to facilitate interest and improve the adherence of patients with chronic conditions such as asthma, diabetes mellitus, and glaucoma [14-16]. In a recent study, an ICT-based telehealth program with voice recognition technology was effective in achieving glycemic control without hypoglycemia in elderly diabetic patients [15]. Given that voice recognition technology can facilitate the gathering of data that are currently difficult to retrieve, as it enables patients, particularly the elderly, to easily access the system and input their data and does not entail additional equipment costs to collect the necessary data, an ICT-based telehealth program incorporating this technology may be a promising tool for improving clinical or laboratory outcomes in chronic HF patients. However, data regarding the role of this novel suite of technologies in the self-management of HF patients are scarce.

With the expectation of enhanced interactivity and active engagement of participants and delivery of tailored interventions, ultimately leading to effective self-care of HF, we developed an ICT-based telehealth program featuring voice recognition technology for HF and sought to explore whether this new program influences the control of sodium intake and quality of life in patients with chronic HF.

## Methods

### Study Design and Population

This study was designed as a prospective single-arm pilot study. The study sample was recruited from consecutive HF patients who were referred to the outpatient clinic of our institute since July 2014. Inclusion criteria were as follows: (1) age ≥18 years; (2) left ventricular (LV) systolic HF diagnosis for >3 months, regardless of etiology according to current guidelines [3,9]; (3) LV ejection fraction (EF) <40% on transthoracic echocardiography of a duration of at least 3 months; (4) New York Heart Association (NYHA) class II or III; and (5) clinical stability including no change in medications for at least the past 4 weeks. Exclusion criteria were the following: (1) hospitalization within 3 months or a condition expected to require hospitalization; (2) a history of acute myocardial infarction or unstable angina within 6 months; (3) a history of significant valvular heart disease requiring surgical or interventional correction; (4) renal replacement therapy; (5) severe hepatic dysfunction defined as aspartate transaminase and alanine transaminase ≥3 times the upper normal limits; (6) malignancy with life expectancy <6 months; (7) childbearing potential or breastfeeding; (8) functional impairment related to severe musculoskeletal or neurological problems; and (9) refusal to participate. The study protocol was in accordance with the Declaration of Helsinki and approved by the institutional review board of our institution. Written informed consent was obtained from all the enrolled subjects.

**Figure 1:**
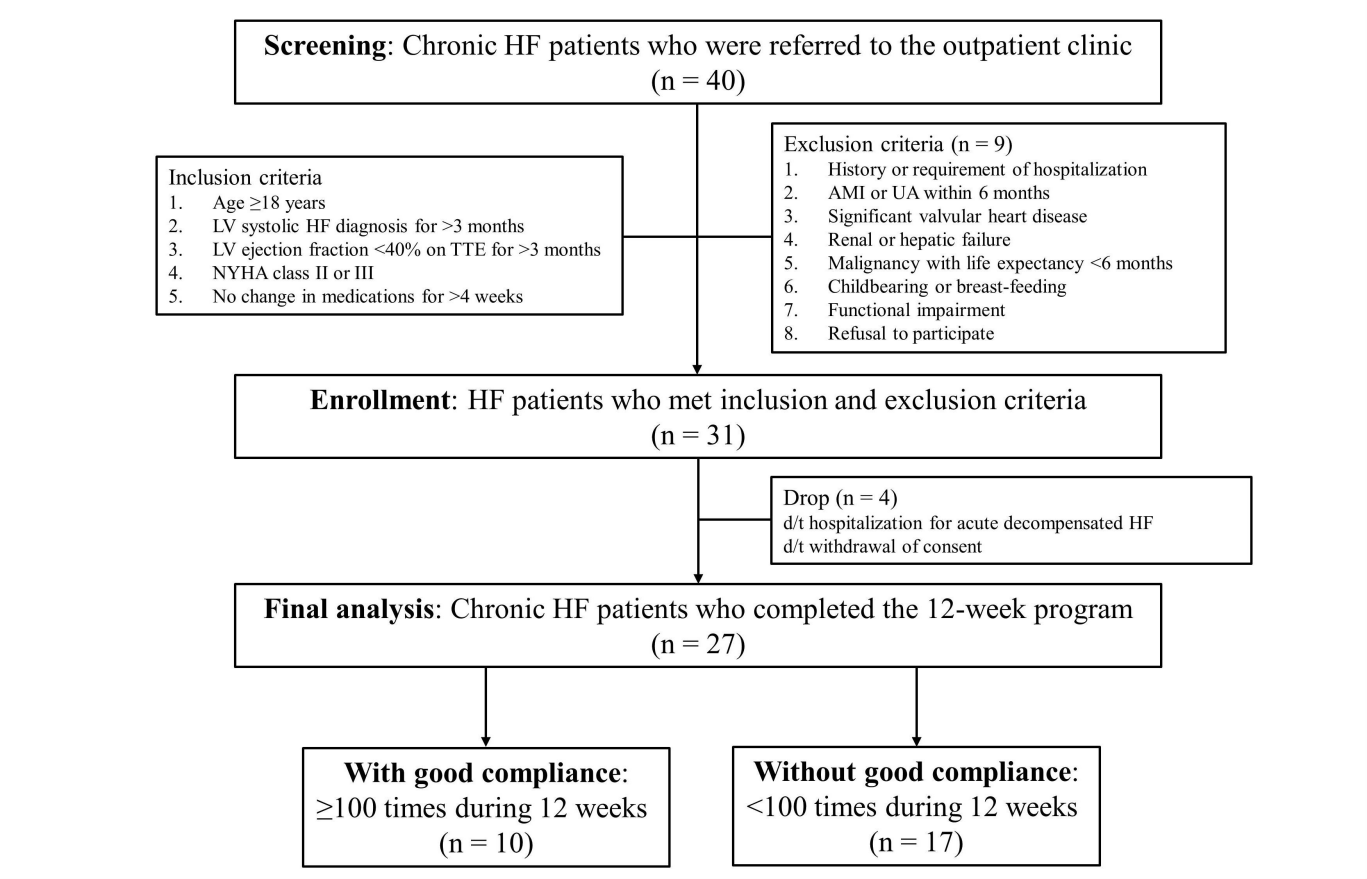
Study flow of ICT-based telehealth program in HF. ICT: information communication technology. HF: heart failure, LV: left ventricular, NYHA: New York Heart Association, TTE: transthoracic echocardiography, AMI: acute myocardial infarction, UA: unstable angina.

Patients were educated regarding the full range of HF self-care behaviors, including diet, exercise, and medication adherence. All participants underwent the following assessments at baseline and after 12 weeks of the ICT-based telehealth program: medical history, physical examination, laboratory tests including urine sodium concentration (uNa) and N-terminal prohormone of brain natriuretic peptide (NT-proBNP), 6-min walk test, NYHA functional class, and Minnesota Living with Heart Failure Questionnaire (MLHFQ) scores [17-19]. The study endpoints included the changes in uNa, MLHFQ scores, NT-proBNP, and 6-min walk distance after 12 weeks of intervention. The adherence to the telehealth program was also analyzed, and a good adherence was defined as the use of the program for 100 times or more during the 12-week intervention period. The study flow is illustrated in Figure 1.

### The ICT-Based Telehealth Program

**Figure 2:**
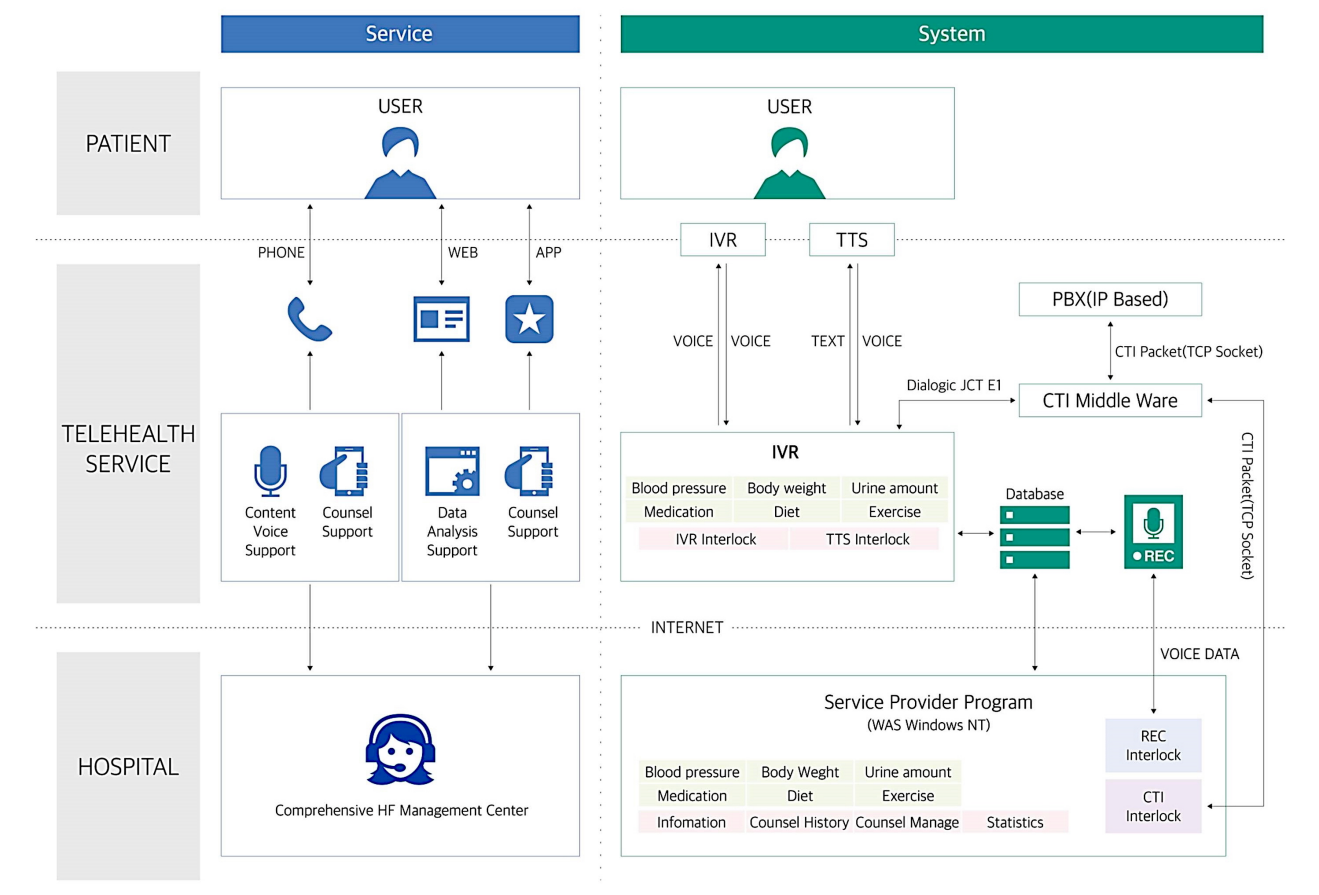
Schematic diagram of the ICT-based telehealth program in HF. ICT: information communication technology, HF: heart failure, TTS: text to speech, IVR: interactive voice response, PBX: private branch exchange, CTI: computer telephony integration, TCP: transmission control protocol, WAS: Web application server, REC: recording, IP: Internet Protocol.

The ICT-based telehealth center comprised multidisciplinary team members, including experienced cardiologists, professional nurses, a dietitian, and computer programmers. Participants were instructed to use the ICT-based telehealth program with voice recognition technology (AIMMED Co Ltd) that was developed for HF patients to provide data and to communicate with clinicians more conveniently through ordinary communication channels via their handheld mobile phone or landline. Patients used their voice or touch-tone telephone keypads to enter essential information for the assessment of HF, including daily weight, diet, exercise, medication adherence, overall symptom change, and home blood pressure. Figure 2 illustrates the schematic diagram of the ICT-based telehealth program.

**Figure 3:**
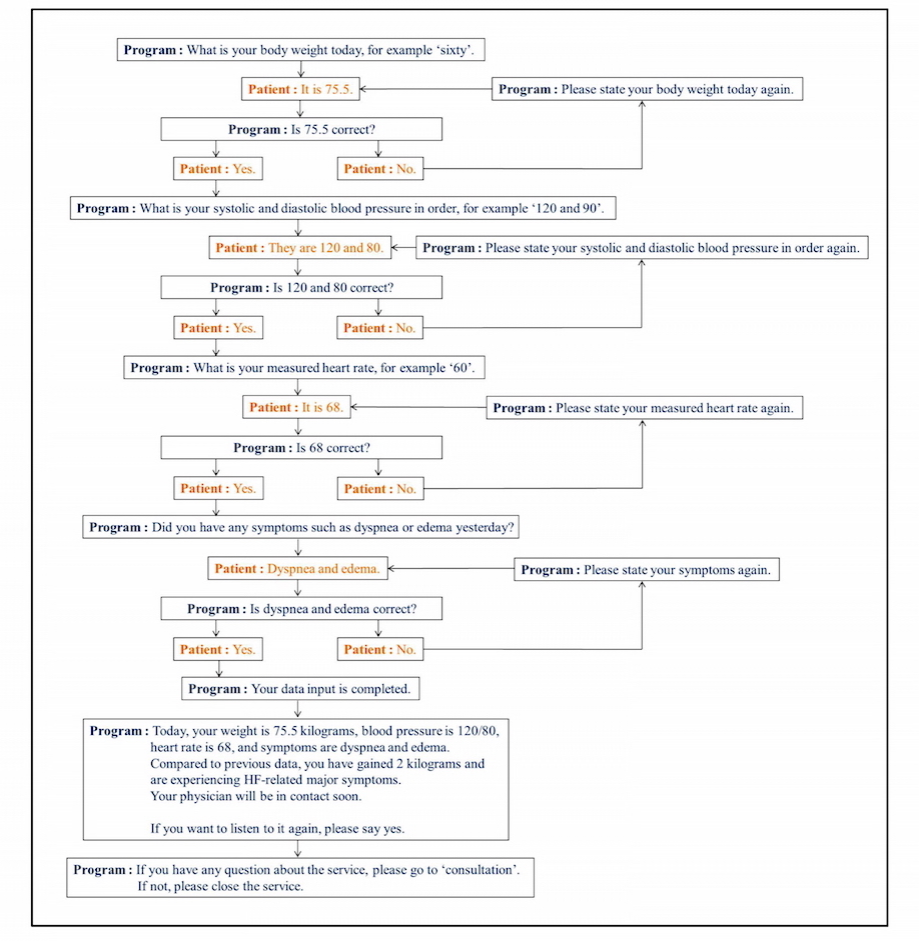
An example of flow diagram for voice recognition program. The voice recognition component of the automatic call and response system enabled the recognition of the patient’s voice data to be input into the ICT-based telehealth program. Patients were requested to speak a pre-set range of inputs, such as body weight, blood pressure, heart rate, and heart failure-related symptoms. Patients were guided by a series of pre-specified verbal questions and examples of possible answers. To ensure the accuracy of the obtained data, the program allowed patients to either confirm or correct the initial extracted data.

Direct voice input of health-related data was performed via landline or mobile phone through the automatic call and response system. The automatic call system was programmed to automatically dial the participants’ registered phone numbers once daily 5 days per week for 12 weeks, and when a person answered the phone, it asked prespecified questions to collect narrative responses. Additionally, patients were also allowed free calls into the system during the study period to collect health-related data from participants who did not answer the call from the automatic call system. Patients, guided by a series of prespecified verbal questions, were requested to speak or to enter a preset range of inputs, including body weight, blood pressure, heart rate, and HF-related symptoms. Examples of possible answers were also presented for patients’ convenience and to reduce the error of the data collection. To further ensure the accuracy of data, the program provided patients the opportunity to either confirm or correct the initial extracted data. An example flow diagram for voice recognition is shown in Figure 3.

A clinical decision support system was implemented to provide immediate tailored feedback by voice or text messages (short message service, SMS) to patients according to a predetermined algorithm following current guidelines [3,20]. When data entries were outside predefined ranges or symptoms were reported, an automatic phone call was made to patients to confirm the data and to their attending physicians and research nurses to notify them of the occurrence of these events. Patients received timely self-care feedback from their physicians and were monitored to ensure resolution. Attending physicians were able to tailor HF management and adjust clinic visit schedules, taking into account the clinical status of each patient. After an out-of-range value occurred, the choice of whether to modify the limits of the range was at the discretion of the physician, who made the decision weighing the individual risk and benefit. Patients were instructed on the use of the website or mobile app created for this study, which provided individually tailored feedback messages based on data entered by the patients (Multimedia Appendix 1). By analyzing the cumulative data of each individual, patients also received personalized guidance on long-term self-care management of HF via the website or as a mobile report. We summarized the functionalities of the ICT-based telehealth program used in our study compared with previous programs in Multimedia Appendix 2.

### Data Collection

Venous blood and urinary samples were obtained from all patients at baseline and after completion of 12 weeks of ICT-based telehealth program. Data including uNa, NT-proBNP, 6-min walk test, and MLHFQ scores were collected to assess the impact of this program on patient outcomes. Specifically, uNa and NT-proBNP were used to assess the adherence to a low-salt diet and the progression of HF, respectively. To assess functional capacity, 6-min walk test was conducted using a standardized protocol between 11 AM to 2 PM on usual medications [21,22]. Briefly, patients were required to perform a 6-min shuttle walk test with markers placed every 25 m. During the 6-min walk test, patients were accompanied by trained research nurses, who were independent of this study, for the safety of participants as well as for the accuracy and reliability of the results. We also used MLHFQ scores to quantify and compare health-related quality of life outcomes before and after the telehealth program [17-19]. The MLHFQ is an established questionnaire, which was developed to measure the subjective influence of HF and HF treatments on an individual’s quality of life, including physical, socioeconomic, and emotional aspects. It comprises 21 items with scores ranging from 0 to 105, and higher scores indicate a lower quality of life in HF patients [17,19].

### Statistical Analysis

Descriptive statistics were used to characterize the study population and measurements. Data were presented as numbers and percentages for categorical variables and mean ± standard error for continuous variables. Differences between continuous variables were compared by the Student *t* test or Mann-Whitney *U* test for independent samples, and those between categorical variables were analyzed by the chi-square test or Fisher exact test, as appropriate. A *P* value of <.05 was considered statistically significant. The Wilcoxon signed-rank test was used to compare values before and after ICT-based telehealth program. In addition, subgroup analysis was also performed according to the adherence to intervention.

## Results

### Baseline Characteristics of Study Population

Of the 40 chronic HF patients who were screened, 31 subjects met the inclusion and exclusion criteria and agreed to participate in this study. Furthermore, 4 patients (4/31, 13%) were dropped from the study because of hospitalization for acute decompensated HF (n=2) and withdrawal of consent (n=2). Two patients hospitalized for acute decompensated HF withdrew within 1 week and 4 weeks of starting the study, respectively, and the cause of HF hospitalization was considered to be a viral infection in both patients. The final analysis was performed in 27 patients (27/31, 87%) who completed the baseline and 12-week measurements. Ten patients (10/27, 37%) showed good adherence to the program, defined as the frequency of its use ≥100 times during the 12-week intervention period. Table 1 describes the baseline characteristics of the total study population and subgroups according to adherence to the intervention. The mean age of the total study population was 63.4 years, and one-third were females (9/27, 33%). Nearly three-fourths had been hospitalized at least once because of HF before the enrollment (20/27, 74%). The number of prior HF hospitalizations per individual was 1.15; 14 patients had 1, 2 patients had 2, and 4 patients had 3 or more previous HF hospitalizations. Before the program started, 70% of patients were already undergoing treatment with diuretics, including mineralocorticoid receptor antagonists (19/27, 70%). There were no changes in HF medications or dosages during the study period. The etiology of 8 patients was ischemia. The baseline mean values of LV ejection fraction, MLHFQ scores, and 6-min walk distance were 30%, 22.2 points, and 408.4 m, respectively. When comparing the baseline characteristics between patients according to adherence to the program, there was no significant difference between the groups. Patients with good adherence tended to have worse baseline parameters, including significantly more frequent hospitalizations related to HF, greater levels of NT-proBNP and uNa, higher MLHFQ scores, and shorter 6-min walk distances, compared with those without (Table 1).

### Approach and Satisfaction of ICT-Based Telehealth Program

Twenty-one patients utilized the program via their mobile phone or landline, and about 59% (16/27) of them also accessed the website. The accuracy of voice recognition was 93% and 95% in patients using mobile phone and landline, respectively. Of the patients in whom the user experience with the voice recognition system could be evaluated, ≥85% patients (23/27) indicated that they were satisfied or neutral with the service provided, as shown in Multimedia Appendix 3. Patients were interested in the following services: blood pressure (27/27, 100%), followed by body weight (26/27, 96%; 951 cases/week), and medication (26/27, 96%; 776 cases/week). Table 2 shows the utilization according to the method and the components accessed.

**Table 1:** Baseline characteristics according to adherence to information communication technology (ICT)–based telehealth program in heart failure (HF).

Characteristics		All patients (N=27)	Patients with good adherence (n=10)	Patients without good adherence (n=17)	*P* values
**Demographics**						
	Age in years, mean ± SE^a^	63.4 ± 1.8	62.1 ± 2.7	64.1 ± 2.4	.60
	Female gender, n (%)	9 (33)	2 (20)	7 (41)	.41
	Height in cm, mean ± SE	161.4 ± 1.4	164.7 ± 1.9	159.4 ± 1.8	.07
	Weight in kg, mean ± SE	63.7 ± 1.9	64.8 ± 2.8	63.1 ± 2.7	.70
	Body mass index in kg/m^2^, mean ± SE	24.4 ± 0.6	23.9 ± 0.4	24.7 ± 0.6	.52
**Clinical history, n (%)**						
	Hypertension	12 (44)	4 (40)	8 (47)	.72
	Diabetes mellitus	6 (22)	3 (30)	3 (18)	.46
	Atrial fibrillation	11 (41)	4 (40)	7 (41)	.95
	Chronic obstructive pulmonary disease	1 (4)	1 (10)	0 (0)	.18
	Chronic kidney disease	10 (37)	4 (40)	6 (35)	.81
	Prior myocardial infarction	9 (33)	5 (51)	4 (24)	.16
	Prior hospitalization due to HF^b^	20 (74)	6 (60)	14 (82)	.20
**Signs and symptoms on enrollment, mean ± SE**						
	Systolic BP^c^, mm Hg	117.3 ± 2.5	116.1 ± 4.3	117.9 ± 3.1	.73
	Diastolic BP, mm Hg	68.4 ± 1.3	68.5 ± 1.4	68.4 ± 1.9	.98
	Heart rate, beats per minute	67.9 ± 2.3	64.4 ± 4.0	69.9 ± 2.8	.26
**Medications on enrollment, n (%)**						
	Diuretics	19 (70)	6 (60)	13 (77)	.42
	ACEI^d^	9 (33)	4 (40)	4 (29)	.68
	ARB^e^	15 (56)	4 (40)	11 (65)	.26
	Beta blocker	12 (44)	4 (40)	8 (47)	>.99
	Calcium channel blocker	4 (15)	1 (10)	3 (18)	>.99
	Spironolactone	19 (70)	7 (70)	12 (71)	>.99
	Digoxin	8 (30)	3 (30)	5 (29)	>.99
**Etiology, n (%)**						
	Ischemic	8 (30)	4 (40)	4 (29)	.37
	Nonischemic	19 (70)	6 (60)	13 (77)	.42
**Laboratory variables, mean ± SE**						
	WBC^f^, × 10^3^/uL	6.4 ± 0.3	6.1 ± 0.5	6.6 ± 0.3	.37
	Hemoglobin, g/dL	13.4 ± 0.3	13.6 ± 0.5	13.3 ± 0.4	.60
	Platelet, × 10^3^/uL	196.7 ± 9.5	178.6 ± 14.3	207.3 ± 12.0	.15
	Sodium, mmol/L	139.6 ± 0.5	140.2 ± 0.7	139.2 ± 0.6	.35
	Potassium, mmol/L	4.6 ± 0.1	4.7 ± 0.2	4.5 ± 0.1	.27
	Chloride, mmol/L	102.9 ± 0.6	104.6 ± 0.8	101.9 ± 0.8	.13
	Total CO_2_^g^, mmol/L	28.5 ± 0.6	27.2 ± 1.2	29.2 ± 0.7	.13
	Calcium, mg/dL	9.4 ± 0.1	9.2 ± 0.2	9.5 ± 0.1	.13
	Phosphate, mg/dL	3.6 ± 0.1	3.5 ± 0.1	3.8 ± 0.1	.13
	Blood urea nitrogen, mg/dL	18.2 ± 1.3	17.7 ± 2.5	18.5 ± 1.5	.78
	Creatinine, mg/dL	1.1 ± 0.1	1.2 ± 0.3	1.0 ± 0.1	.35
	GFR^h^, mL/min/1.73 m^2^	69.8 ± 3.6	70.6 ± 7.7	69.3 ± 3.8	.88
	NT-proBNP^i^, pg/mL	288.6 ± 65.7	300.2 ± 108.1	281.7 ± 85.2	.90
	Urine sodium, mmol/L	91.9 ± 0.5	103.1 ± 9.2	85.4 ± 10.5	.26
	LV^j^ ejection fraction, %	30.3 ± 1.3	30.7 ± 2.4	30.1 ± 1.5	.81
Quality of life data, mean ± SE	MLHFQ^k^ score	22.2 ± 3.2	27.5 ± 6.4	19.0 ± 3.3	.20
					
Functional status data, mean ± SE	6-min walk distance, m	408.4 ± 16.1	404.0 ± 30.1	411.0 ± 19.2	.84
					

^a^SE: standard error.

^b^HF: heart failure.

^c^BP: blood pressure.

^d^ACEI: angiotensin-converting enzyme inhibitor.

^e^ARB: angiotensin receptor blocker.

^f^WBC: white blood cell.

^g^CO_2_: carbon dioxide.

^h^GFR: glomerular filtration rate.

^i^NT-proBNP: N-terminal prohormone of brain natriuretic peptide.

^j^LV: left ventricular.

^k^MLHFQ: Minnesota Living with Heart Failure Questionnaire.

**Table 2:** Data on utilization of information communication technology (ICT)–based telehealth program in heart failure (HF).

Data on utilization		Patients accessed, n (%)	Frequency of access per week
**Type of access method**				
	Mobile phone or landline	21 (77.8)	2297
	ACS^a^/ARS^b^	10 (37.0)	139
	TTS^c^	18 (66.7)	848
	Website access	16 (59.3)	433
**Type of accessed component**				
	Body weight	26 (96.3)	951
	Diet	24 (88.9)	419
	Exercise	25 (92.6)	657
	Medication	26 (96.3)	776
	Overall symptom change	22 (81.5)	651
	Blood pressure	27 (100.0)	2812

^a^ACS: automatic call system.

^b^ARS: automatic response system.

^c^TTS: text to speech.

### Changes After Using ICT-Based Telehealth Program

**Figure 4:**
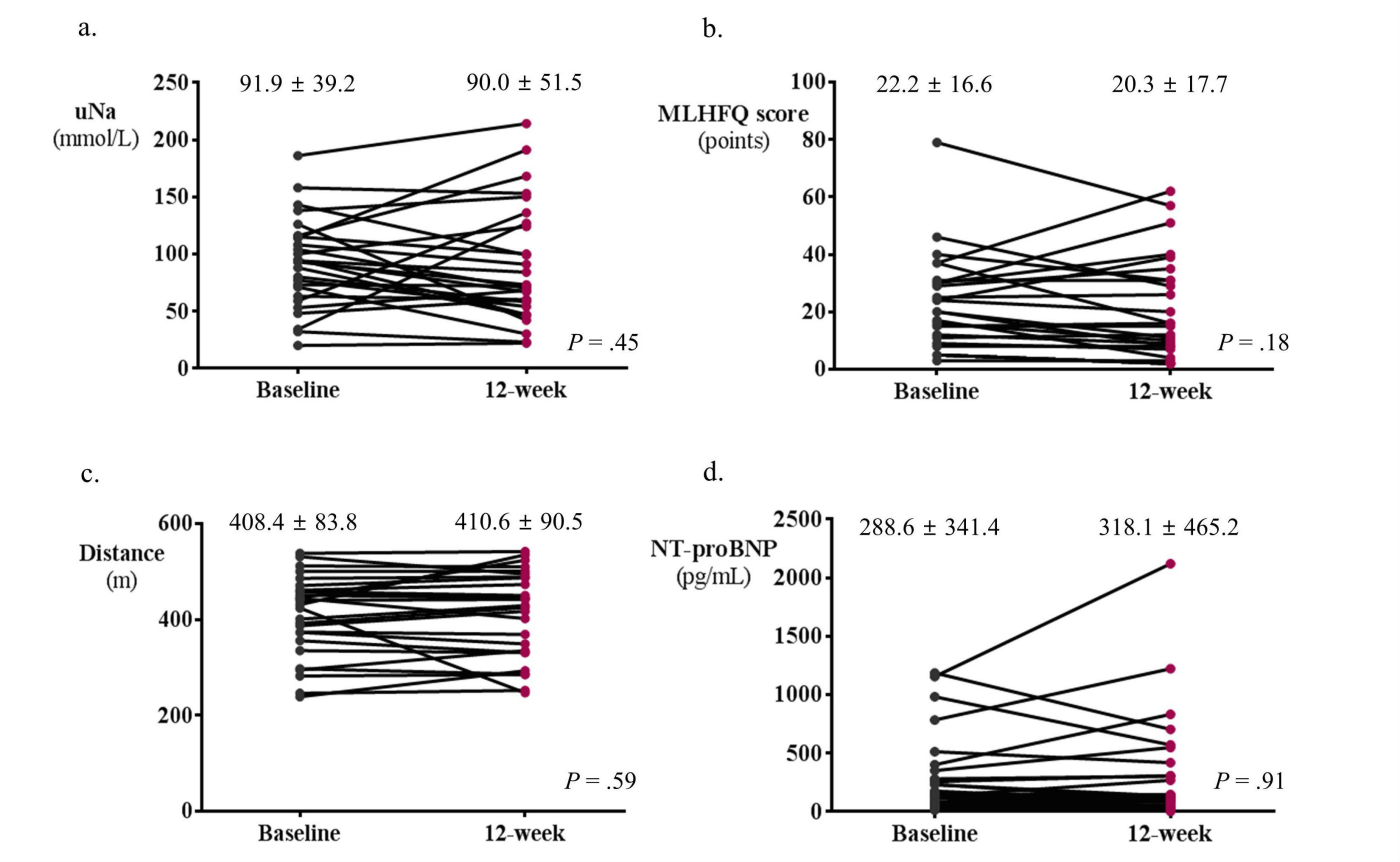
Changes in major outcomes over time by ICT-based telehealth program in total study population. There was no significant changes in uNa (a), MLHFQ scores (b), 6-minute walk distance (c), and NT-proBNP level (d) after 12 weeks of ICT-based telehealth program. ICT: information communication technology, HF: heart failure, NT-proBNP: N-terminal prohormone of brain natriuretic peptide, MLHFQ: Minnesota Living with Heart Failure Questionnaire.

**Figure 5:**
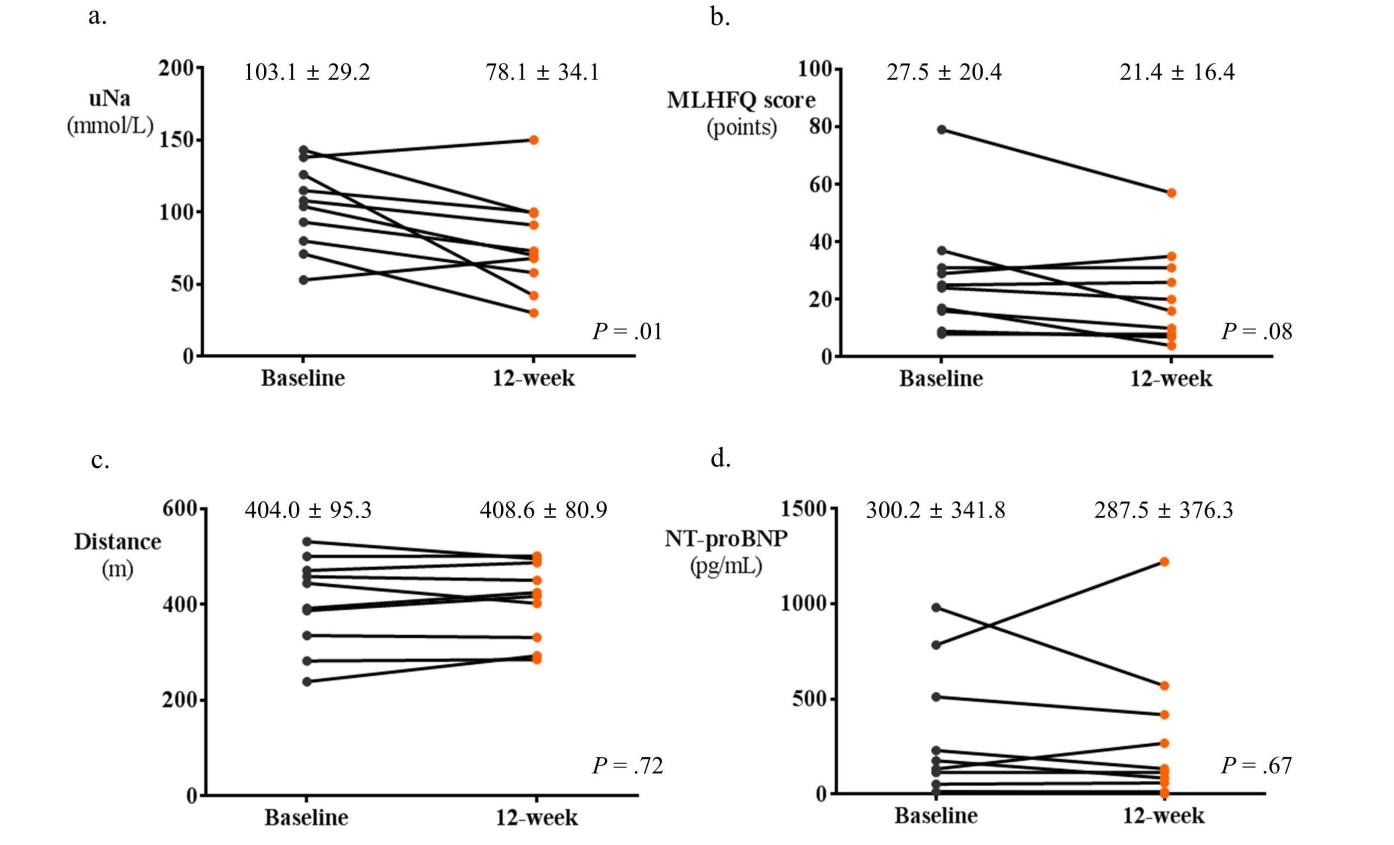
Changes in major outcomes over time by information communication technology (ICT)–based telehealth program in patients with good adherence. In patients with good adherence, uNa significantly decreased after 12 weeks of intervention (a), whereas MLHFQ scores marginally decreased (b). There were no changes in 6-minute walk distance (c) and NT-proBNP level (d). NT-proBNP: N-terminal prohormone of brain natriuretic peptide, MLHFQ: Minnesota Living with Heart Failure Questionnaire.

**Figure 6:**
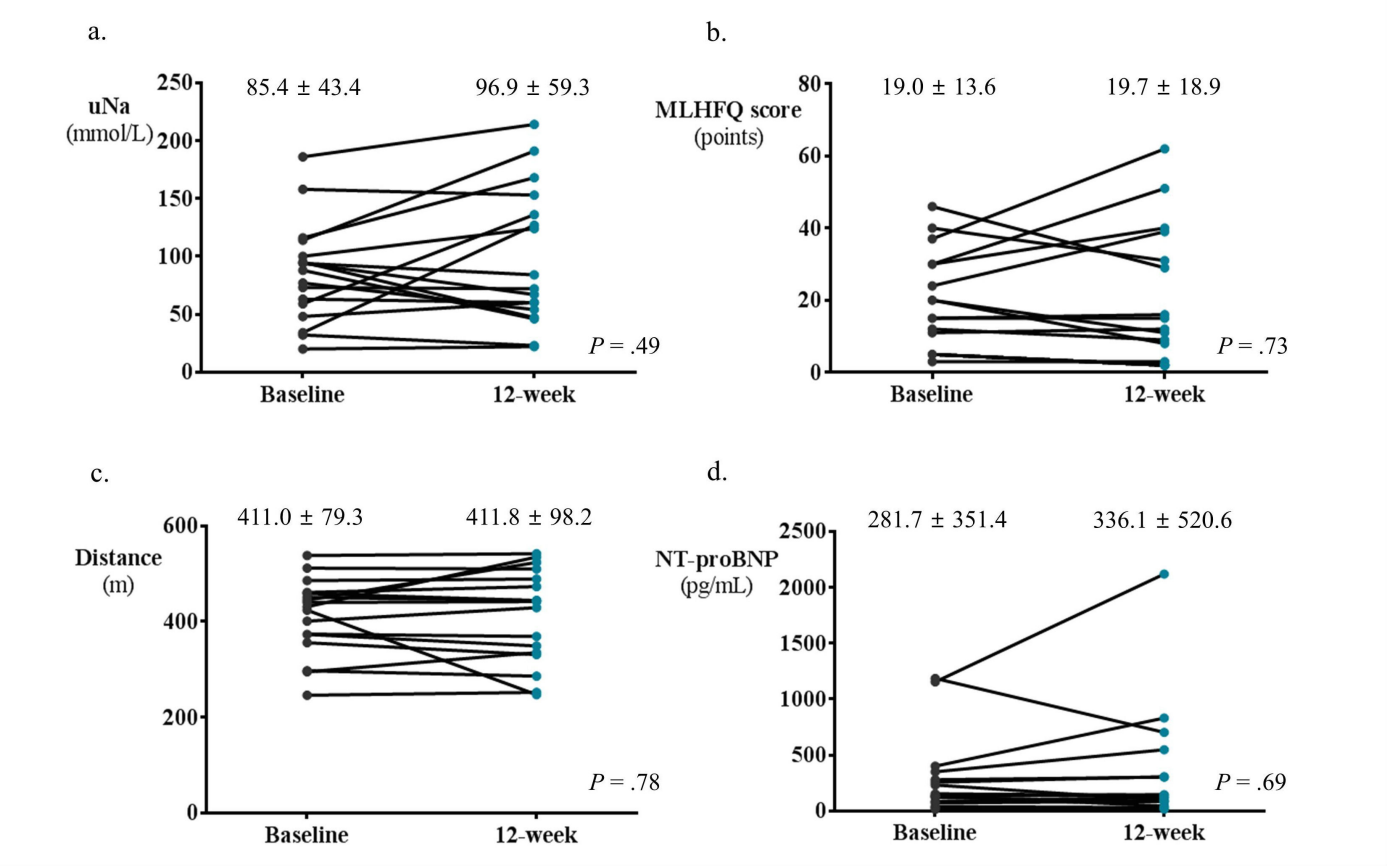
Changes in major outcomes over time by information communication technology (ICT)–based telehealth program in patients without good adherence. In patients without good adherence, no significant changes were observed in laboratory and functional outcomes after 12 weeks of ICT-based telehealth program (a-d). NT-proBNP: N-terminal prohormone of brain natriuretic peptide, MLHFQ: Minnesota Living with Heart Failure Questionnaire.

In the total study population, there was no significant interval change in laboratory and functional outcome variables after 12 weeks of ICT-based telehealth program (Figure 4): uNa (91.9 to 90.0; *P*=.45), MLHFQ scores (22.2 to 20.3; *P*=.18), 6-min walk distance, (408.4 to 410.6; *P*=.59), and NT-proBNP (288.6 to 318.1; *P*=.91). When we stratified patients into two groups by adherence to the ICT-based telehealth program, there was a significant reduction in the value of uNa (103.1 to 78.1; *P*=.01) and a marginal decrease in the MLHFQ scores (27.5 to 21.4; *P*=.08) in HF patients with good adherence (Figure 5). However, no significant changes were observed in those without good adherence regarding these variables (Figure 6). On the other hand, 6-min walk distance neither significantly improved in patients with good adherence nor in those without (Figure 6). No significant change in NT-proBNP was observed in both groups of patients (Figures 5 and 6). The MLHFQ scores decreased after 12 weeks of ICT-based telehealth program in 18 patients (18/27, 67%). When we assessed the change in the subscores of physical (8 items), emotional (5 items), and socioeconomic (8 items) dimensions of the MLHFQ, although statistically insignificant, patients with good adherence showed numerically greater improvements in all dimensions. Specifically, the improvement in the total score of MLHFQ was largely driven by the decrease in emotional dimension measures among three quality of life dimensions (94%, 17/18 patients). The change in emotional domain scores was −2.1 in patients with good adherence and −0.2 in those without (*P*=.48). Results in terms of physical dimension measures revealed that the change was −2.5 in patients with good adherence and 0.35 in those without (*P*=.13). Regarding socioeconomic dimension measures, the change was −1.5 in patients with good adherence and 0.5 in those without (*P*=.11).

## Discussion

### Principal Findings

This study is a prospective pilot study examining the possibility of ICT-based telehealth program with voice recognition technology in HF and building up the foundation for further large-scale randomized controlled studies. Our study demonstrated that chronic HF patients with good adherence to ICT-based telehealth program showed improvement in uNa and in symptoms evaluated by the MLHFQ scores. To the best of our knowledge, this is the first study to implement this novel technology to improve self-care of HF. Our study may pave the way for further large-scale clinical outcome trials to determine the efficacy and feasibility of ICT-based interventions for long-term management of HF patients.

### Impact of ICT-Based Telehealth Program in HF on Urine Sodium Concentration

The key characteristics of HF include an increase in sodium avidity and a tendency toward congestion [23,24]. Therefore, the maintenance of sodium and water balance is a critical element of HF management, and among several noninvasive methods proposed to assess this balance, uNa has been increasingly used as a simple, inexpensive, and noninvasive indicator for the dietary sodium intake [25-29]. Specifically, it is well established that the urinary sodium excretion is precisely regulated to match dietary sodium intake [25,30]. Although the assessment of 24-hour urine sodium excretion is considered the gold standard method, the measurement of spot uNa could provide an approximate estimate of sodium excretion, with increased convenience and low cost [25,29,31-34]. On the other hand, a previous study demonstrated that spot uNa was reduced by decongestive therapy for advanced HF, which might provide insightful information to titrate the diuretic dose [35,36]. Hence, in general, decreased uNa in HF patients can be a result of low salt intake or effective diuretic therapy [35]. In this study, HF patients with good adherence to ICT-based telehealth program showed reduced uNa after 12 weeks of intervention. Given that patients were clinically stable without any evidence of acute destabilized HF and change in diuretic dose, decreased uNa might be a reflection of better control of dietary salt intake. As recent data emphasize nonpharmacological as well as pharmacological interventions for HF because of its progressive nature [37], an ICT-based telehealth program can be a useful tool for improving self-care and, potentially, prognosis in HF patients [37].

### Impact of ICT-Based Telehealth Program in HF on the MLHFQ Scores

Improving health-related quality of life is not only a major treatment goal in HF but also an effective surrogate marker of appropriate HF therapy and improved prognosis in HF patients [17,38-40]. Among various methods of assessing health-related quality of life, we used the MLHFQ, which has been extensively validated in HF patients [18,19,38,40,41]. In our study, the mean MLHFQ scores numerically decreased by about 2 points in all patients (*P*=.18) and by 6.1 points in patients with good adherence (*P*=.08) after 12 weeks of ICT-based telehealth program. Although there is no absolute cutoff for the MLHFQ scores to discriminate between normal and impaired quality of life, a decrease of more than 5 points is considered as a clinically important change [42,43]. Previous studies have suggested that the changes in the MLHFQ scores can translate into differences in clinical outcomes of HF patients. For example, EPICAL trial showed that a 10-point decrement in the MLHFQ scores was associated with a 23% decrease in mortality and a 31% decrease in readmission for HF [17]. Hoekstra et al [42] also reported that each 10-point increment in the MLHFQ scores was associated with a 7% increase in mortality. Given that the short duration of intervention in this study led to a 6.1 point decrease in the MLHFQ scores among patients with good adherence, an ICT-based telehealth program seems promising to enhance quality of life and consequently survival of HF patients. Since the MLHFQ comprises three subscores (physical, emotional, and socioeconomic dimensions), we also compared the changes in each of the subscores between groups. In this analysis, the reduction in the MLHFQ scores mostly stemmed from the improvement in emotional aspects in patients with good adherence, although the between-group difference was not significant. These findings suggest that intense monitoring and automated feedback using an ICT-based telehealth program can play a pivotal role in providing emotional support for HF patients. Considering the close relationship between emotional health and hard clinical outcomes in HF [44,45], ICT-based telehealth program has the potential to improve the prognosis of HF patients. The improvement in physical subscores was also numerically greater in patients with good adherence without reaching statistical significance. Considering that patients with good adherence to ICT-based telehealth program showed reduced uNa, a surrogate marker of better control of sodium intake, it seems plausible that this program can also be helpful in improving the physical aspects of quality of life in HF patients. A future study with a large sample size is needed to validate these findings.

### Importance of Adherence in Self-Care Management

Previous studies demonstrated that self-care management using telemonitoring systems did not significantly improve hard endpoints in HF patients, such as all-cause death or HF hospitalization [46,47]. The low rate of adherence to self-care management programs has been suggested as one of possible reasons for these suboptimal results. In the tele-HF trial, only 55% of study patients utilized the telemonitoring system at least 3 times per week during 6 months and, furthermore, 14% of patients never used this system during the study period [46]. In our study, 23 of 27 patients (85.2%) utilized ICT-based telehealth program at least 3 times per week during 12 weeks, and there were no patients who did not use the program. Furthermore, 10 of 27 patients (37.0%) used the ICT-based telehealth program ≥100 times during the 12-week period, indicating the use of this program more than once daily. These patients with good adherence to the program showed the improvement in sodium intake and quality of life. This improved adherence of patients may be partially due to the enhanced integration of ICT-based telehealth program into their daily lives, with the easier use of such systems.

### User Experience With Voice Recognition Component of the Technology

The user experience was positive with ≥85% patients indicating satisfaction or neutral response with the voice recognition system provided. This favorable user experience might have been achieved by the acceptable success rate of voice recognition in our study, considering that 95% accuracy is nearly on par with that of human speech recognition. In this study, however, the accuracy of voice recognition was affected by adverse conditions, including the use of nonstandard speech, voice alteration during walking, and the presence of substantial background noise. Specifically, the success rate of voice recognition was 91% and 86% for the patients who used nonstandard language and for those who were walking during the process of voice recognition, respectively. Furthermore, the success rate was 87% in settings with increased background noise level, such as when using public transportation. Hence, the user experience can be further optimized by improving the accuracy of voice recognition under these unfavorable circumstances.

### Implications of Using ICT-Based Telehealth Program With Voice Recognition Technology on Study Endpoints

HF patients performing and maintaining self-care management behaviors, such as control of sodium intake (uNa), is one practical implication of this technology from a health care perspective. Although future studies with larger number of HF patients are warranted, this technology also has the potential to improve health-related quality of life (MLHFQ scores). Current implications for improving functional capacity (6-min walk test) or HF severity (NT-proBNP) are limited. Further studies are also needed to determine whether the use of this technology has workforce implications in the management of HF patients.

### Study Limitations

This study has several limitations that should be considered. First, our sample size was small and the duration of intervention was short. This limitation made it difficult to show the benefit of the ICT-based telehealth program for clinical outcomes in HF patients, especially given the chronic and progressive nature of HF. However, in the case of pilot studies, sample sizes of 10 to 30 are generally considered large enough to test the null hypothesis and small enough to ignore weak treatment effects [47]. Furthermore, even though our study population largely comprised patients with moderate to severe systolic dysfunction (LVEF <40%) whose mortality is substantial (25.6%-41.7%) [48], there was no mortality and only 2 patients withdrew because of hospitalization for acute decompensated HF at the early phase of the study. Notably, the most likely cause of HF exacerbation in these patients was viral infection, not a high-salt diet or poor adherence to medication. In addition, there were no rehospitalizations or emergency department visits for HF after 4 weeks of intervention in our study participants, approximately 75% of whom had a history of prior HF hospitalizations, suggesting that clinical outcomes can be improved by applying this strategy to HF patients. Considering that a prior history of HF hospitalizations has been reported as a risk factor for repeat hospitalizations in HF patients [49,50], our findings suggest that clinical outcomes can be improved by the appropriate application of ICT-based telehealth program in HF patients, although larger studies with longer follow-up are definitely warranted. Second, the definition of good adherence was arbitrary. We assumed that the total frequency of program use of more than 100 times during the follow-up of 12 weeks could be regarded as accessing the system at least once per day. Third, we did not have data on baseline education level, including literacy, health care literacy, digital literacy, and numeracy skills of the study population, which can be critical components for successful technology uptake [51]. Fourth, although spot uNa provides an approximate estimate of sodium excretion, it has limited usefulness as an indicator of long-term dietary behavior change because of its short-term relevance. Thus, a future study using a more accurate surrogate marker, such as 24-hour urine sodium excretion, is warranted to more clearly discern the beneficial effect of ICT-based telehealth program on sodium intake. Finally, because of the lack of an appropriate control arm, study findings should be interpreted with caution. A prospective randomized study comparing an ICT-based telehealth program with conventional HF care is necessary to define the true clinical benefit from this therapeutic strategy in HF patients.

### Conclusions

Short-term application of ICT-based telehealth program in HF demonstrated the potential to improve control of sodium intake and quality of life in chronic HF patients with good adherence to this program. Our results suggest that an ICT-based telehealth program can be an effective strategy to achieve better patient care and clinical outcomes in HF.

## Appendix

Multimedia Appendix 1 The ICT-based telehealth program website.

Multimedia Appendix 2 Comparison of characteristics of ICT-based telehealth program with voice recognition technology and previous programs.

Multimedia Appendix 3 Patient satisfaction with the voice recognition component of the technology.

## Abbreviations

ACEI: angiotensin-converting enzyme inhibitor
ACS: automatic call system
ARB: angiotensin receptor blocker
ARS: automatic response system
BP: blood pressure
CO2: carbon dioxide
EF: ejection fraction
GFR: glomerular filtration rate
HF: heart failure
ICT: information communication technology
LV: left ventricular
MLHFQ: Minnesota Living With Heart Failure Questionnaire
NT-proBNP: N-terminal prohormone of brain natriuretic peptide
NYHA: New York Heart Association
SE: standard error
SMS: short message service
TTS: text to speech
uNa: urine sodium concentration
WBC: white blood cell
