# Supplementary material for: Impact of a Telehealth Program With Voice Recognition Technology in Patients With Chronic Heart Failure: Feasibility Study
Source: JMIR Mhealth Uhealth. 2017 Oct 2;5(10):e127. doi: 10.2196/mhealth.7058 (PMC5643844; doi:10.2196/mhealth.7058)
Supplement: Multimedia Appendix 1 [file mhealth_v5i10e127_app1.pdf]

Multimedia Appendix 1. The ICT-based telehealth program website

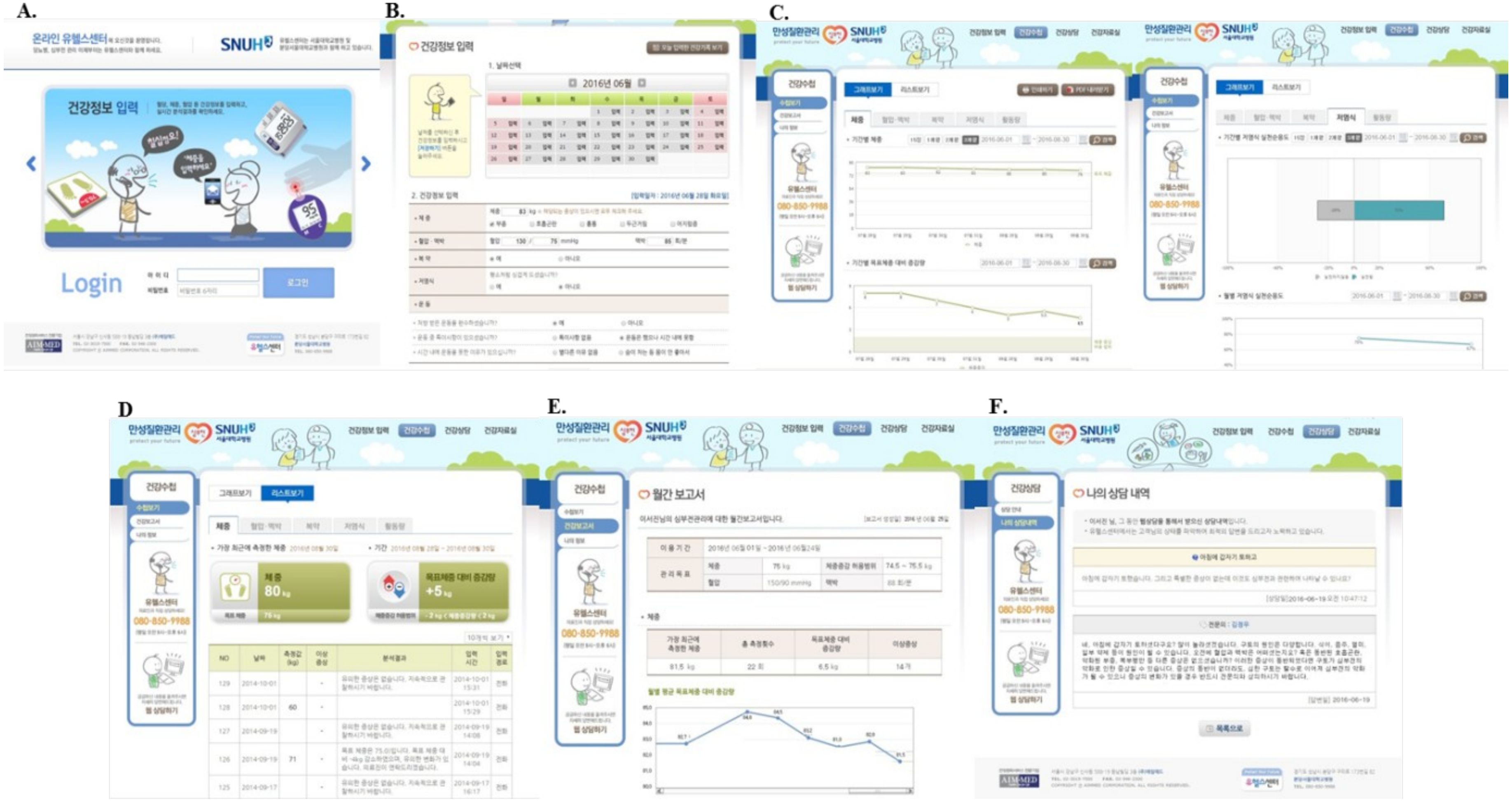

The website was created as an online platform for the ICT-based telehealth program. After logging in to the website (A), the latest data were provided to the patients. Patients were asked to enter their clinical and behavioral data, including body weight, blood pressure, heart rate,

medications, salt intake, and exercise, into the data entry page (B). Entered data were analyzed comprehensively and patient-specific recommendations were provided to the participants by the clinical decision support system (C-D). Monthly reports were generated to provide insight into the trend of changes in clinical and behavioral data of each individual patient (E). Additionally, when patients submitted a question regarding the individualized health consult section, experts provided detailed answers and practical recommendations (F).

ICT = information communication technology.
