# Supplementary material for: Impact of a Telehealth Program With Voice Recognition Technology in Patients With Chronic Heart Failure: Feasibility Study
Source: JMIR Mhealth Uhealth. 2017 Oct 2;5(10):e127. doi: 10.2196/mhealth.7058 (PMC5643844; doi:10.2196/mhealth.7058)
Supplement: Multimedia Appendix 2 [file mhealth_v5i10e127_app2.pdf]

Multimedia Appendix 2. Comparison of characteristics of ICT-based telehealth program with voice recognition technology and previous programs

	New ICT-based telehealth program	Previous telehealth programs
**Technical aspect**	• Minimal technical requirement (mobile phone or landline)<br>• Voice recognition data entry<br>• Integration into health care processes permitting immediate and effective decision making and feedback	• Requirement of auxiliary devices for sensing and/or transmitting data<br>• Typed data entry<br>• Provision of telemonitoring support only
**Clinical implications**	• Potential for improvement in patient adherence<br>• Potential for timely interventions	• Potential for improvement in patient adherence<br>• Potential for close monitoring, but without timely actions by health care provider
**User-perspectives**	• Low barrier for using the program, even for elderly or illiterate users	• Need for further education and training of users, particularly the elderly or the illiterate, to operate the programs
**Socio-economic impact**	• Elimination of additional costs arising from purchase of auxiliary devices<br>• Reduction of healthcare costs related to hospitalizations	• Reduction of healthcare costs related to hospitalizations

ICT = information communication technology.
