# Supplementary material for: Impact of a Telehealth Program With Voice Recognition Technology in Patients With Chronic Heart Failure: Feasibility Study
Source: JMIR Mhealth Uhealth. 2017 Oct 2;5(10):e127. doi: 10.2196/mhealth.7058 (PMC5643844; doi:10.2196/mhealth.7058)
Supplement: Multimedia Appendix 3 [file mhealth_v5i10e127_app3.pdf]

Multimedia Appendix 3. Patient satisfaction with the voice recognition component of the technology

Supplementary Figure 1.

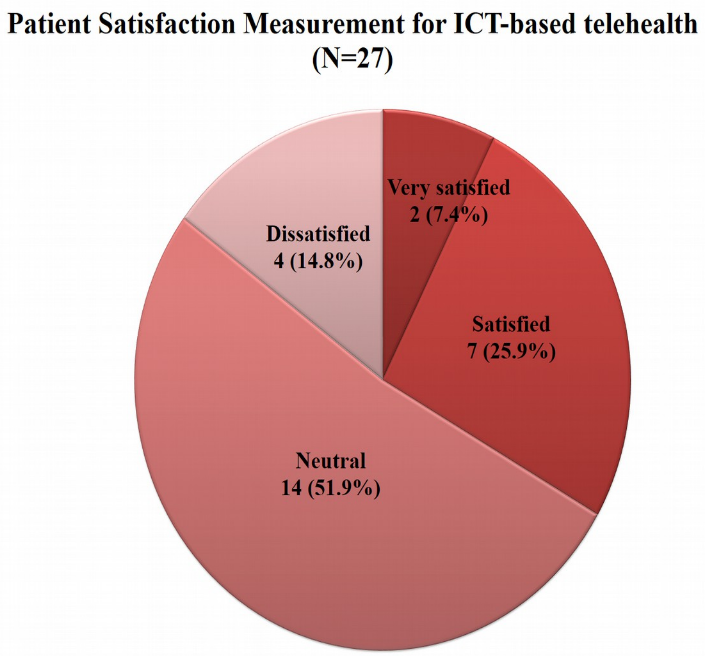

Of 27 patients in whom the user experience with the voice recognition system could be evaluated, 2 patients (7.4%) were “Very Satisfied”, 7 (25.9%) were “Satisfied”, and 14 (51.9%) were “Neutral”.
